# Uncarilic Acid and Secouncarilic Acid, Two New Triterpenoids from *Uucaria sessilifructus*

**DOI:** 10.3390/molecules18089727

**Published:** 2013-08-14

**Authors:** Mao-Juan Zhang, Bing Liu, Shang-Gao Liao, You-Kai Xu, De-Qiang Feng, Kai-Long Ji, Yan Li

**Affiliations:** 1 Key Laboratory of Tropical Plant Resource Science, Xishuangbanna Tropical Botanical Garden, Chinese Academy of Sciences, Mengla 666303, Yunnan, China; 2 College of Traditional Chinese Medicine, Yunnan University of Traditional Chinese Medicine, Chenggong, Kunming 650200, Yunnan, China; 3 Engineering Research Center for the Development and Application of Ethnic Medicines and TCM, School of Pharmacy, Guiyang Medical College, 9 Beijing Road, Guiyang 550004, Guizhou, China; 4 State Key Laboratory of Phytochemistry and Plant Resources in West China, Kunming Institute of Botany, Chinese Academy of Sciences, Kunming 650204, Yunnan, China; 5 Graduate University of Chinese Academy of Sciences, 19 Yuquan Road, Beijing 100049, China

**Keywords:** *Uncaria sessilifructus*, uncarilic acid, secouncarilic acid, triterpenoids

## Abstract

Two new compounds, the 6-oxo oleanane-type triterpenoid uncarilic acid, and its 5,6-secotriterpenoid derivative, secouncarilic acid, were isolated from the hooks and stems of *Uucaria sessilifructus* together with seven known ursane-type triterpenoids. Uncarilic acid is the second 6-oxo oleanane-type triterpenoid ever reported, while secouncarilic acid is the first oleanane-type 5,6-secotriterpenoid. A plausible biosynthetic pathway from uncarilic acid to secouncarilic acid was also postulated. The inhibitory activities of all the nine compounds against LPS-induced nitric oxide production in RAW264.7 macrophages were evaluated.

## 1. Introduction

*Uncaria* (Rubiaceae) is a genus of 34 species mainly distributed in the tropical regions such as southern Asia, Africa and South America [1]. As one of the sources of the Chinese drug “Gou-teng”, *Uucaria sessilifructus* Roxb. has been used by the Chinese people for the treatment of hypertension, headache and fever [2], and by the Red-headed Yao people in Jinping of Yunnan Province of China to treat fear, neurotic disorders, high blood pressure, giddiness, bellyache, hysteritis, rheumatoid arthritis, arthritis, hemiplegia, sciatica, injuries from falls, and ulcers [3]. Aside from alkaloids and flavonoids, species of the genus *Uncaria* also contain a variety of pentacyclic triterpenoids, typically derived from ursolic, oleanolic, or quinovic acid [4,5]. In *U. sessilifructus*, a total of fifteen pentacyclic or tetracyclic oxindole alkaloids were identified prior to Philipson’s 1978 review of *Uncaria* alkaloids [6]. The chemical composition of its volatile oil was also reported [7]. Herein, the non-alkaloid components from the hooks and stems of *U. sessilifructus* were investigated for the first time. As a result, two new oleanane-type triterpenoids named uncarilic acid (**1**) and secouncarilic acid (**2**) were isolated together with seven ursane-type triterpenoids **3**–**9** and their inhibitory activities against nitric oxide production in LPS-activated RAW264.7 macrophages were measured. A plausible biosynthetic pathway from **1** to **2** was also postulated.

## 2. Results and Discussion

Compound **1** was obtained as a white amorphous powder. Its molecular formula was deduced to be C_30_H_46_O_5_ from the HREI-MS (*m/z* 486.3345, [M]^+^; calcd. for 486.3356) and confirmed by ^13^C-NMR and DEPT spectra (Table 1). The IR spectrum of **1** exhibited characteristic bands at 3,441 (OH), 1702 (C=O), 1,630 (olefinic C=C) cm^−1^. The ^1^H-NMR of **1** recorded at 500 MHz exhibited seven singlet methyls at *δ* 0.80, 0.91, 0.93, 0.97, 1.01, 1.18 and 1.43, one broad singlet at *δ* 3.13, signals assignable to two methine protons bearing a hydroxyl group at *δ* 3.11 (dd, *J* = 4.0, 11.2 Hz) and 3.27 (br d, *J* = 2.9 Hz) and one signal of an olefinic proton at *δ* 5.35 (br t). In the ^13^C-NMR spectrum, signals corresponding to 28 of the 30 carbons were easily recognized as seven methyl carbons, eight *sp*^3^ methylenes, five *sp*^3^ methines (two oxygenated), five *sp*^3^ quaternary carbons, two *sp*^2^ carbons of a trisubstituted olefin (*δ* 144.6 and 124.2), and one ketone carbon (*δ* 215.9). The missing signals for the remaining two carbons urged us to conduct 2D NMR experiments. In the HSQC spectrum, a carbon signal at *δ*_C_ 49.3 that was buried in the solvent residue signals was detected through its correlation with the proton signal at *δ*_H_ 2.34, The signal was further assigned to C-9 on the basis of the HMBC correlations H-9/C-25and H-7/C-9,H_3_-26/C-9 (at 600/150 MHz in CD_3_OD). Further observation of the carbon signal at *δ* 48.1 in the ^13^C-NMR spectrum recorded in CDCl_3_ (at 125 MHz) also supported this assignment. Although not very strong, the observation of correlations between a weak carbon signal at *δ* 184.6 (C-28) and H_2_-16 in the HMBC spectrum (at 600/150 MHz in CD_3_OD) suggested the presence of a carboxylic acid group at C-17. The above data of **1** were quite similar to those of 3*β*,19*α*,23-trihydroxy-6-oxo- olean-12-en-28-oic acid (**1a**) [8], the distinct difference being the replacement of the 23-hydroxymethyl by a tertiary methyl and the absence of a 28-carboxylic group. The HMBC correlations from the two methyl signals at *δ*_H_ 1.01 (CH_3_-23) and 1.18 (CH_3_-24) to the oxymethine carbon at *δ* 79.2 (C-3), the quaternary carbon at *δ* 38.6 (C-4), and the methine carbon at *δ* 66.5 (C-5) and the cross-peaks between the two methyls also supported attachment of the two methyls at C-4. Furthermore, HMBC correlations from H-5 (*δ* 2.26) and H_2_-7 (*δ* 2.59 and 1.80, each d) to the ketone carbon (*δ* 215.9) indicated that the ketone was located at C-6. The *α*-orientation of H-3 is also supported by its ROESY correlations with H-5. 

**Table 1:** ^1^H- and ^13^C-NMR of **1** and **2**.

No.	1					2	
	*δ*_H_, mult. (*J* in Hz)	*δ*_C_, mult.				*δ*_H_, mult. (*J* in Hz)	*δ*_C_, mult.
	500MHz in CD_3_OD	125 MHz in CD_3_OD	150 MHz in CD_3_OD	150 MHz in CDCl_3_		500 MHz in CD_3_OD	125 MHz in CD_3_OD
1	*α* 1.31, *β* 1.71 m	40.0 t	40.2 t	38.9 t		*α* 2.09 dd (15.1, 8.9), *β* 1.22 m	32.9 t
2	*α* 1.57, *β* 1.63 ^a^	27.4 t	27.6 t	26.8 t		*α* 2.26, *β* 1.68 ^a^	26.2 t
3	3.11 dd (4.0, 11.2)	79.2 d	79.3 d	78.9 d		4.94 t (9.1)	69.3 d
4		38.6 s	38.8 s	37.6 s			56.9 s
5	2.26 s	66.5 d	66.6 d	65.5 d			220.9 s
6		215.9 s	216.0 s	212.7 s			177.1 s
7	*α* 2.59, *β* 1.80 d (12.4)	52.1 t	52.2 t	50.9 t		*α* 2.52, *β* 2.30 d (19.1)	42.2 t
8		47.9 s	48.1 s	46.6 s			45.2 s
9	2.34 dd (7.3, 10.4)	49.3^a^	49.3 d ^c^	48.1 d		3.49 dd (6.5, 10.9)	37.4 d
10		44.4 s	44.6 s	42.3 s			52.8 s
11	*α* 2.08, *β* 1.99 m	25.1 t	25.2 t	24.2 t		*α* 2.29, *β* 2.19 ^a^	27.3 t
12	5.35 br t (3.5)	124.2d	124.4 d	124.8 d		5.47 br t (3.6)	125.4 d
13		144.6 s	144.7 s	142.2 s			144.8 s
14		43.0 s	43.1 s	41.8 s			46.3 s
15	*α* 0.88, *β* 1.68 ^a^	29.5 t	29.7 t	28.1 t		*α* 1.05 m, *β* 1.67 ^a^	31.1 t
16	*α* 2.25, *β* 1.62 ^a^	28.6 t	28.7 t	27.7 t		*α* 2.16, *β* 1.65 ^a^	29.1 t
17		46.5 ^a^	47.2 s	45.3 s			47.2 d
18	3.13 br s	45.5 d	45.6 d	43.6 d		3.16 br s	46.7 d
19	3.27 br d (2.9)	82.7 d	82.4 d	81.8 d		3.28 d (3.7)	82.4 d
20		36.1 s	36.3 s	34.9 s			36.2 s
21	*α* 1.74, *β* 0.98 ^a^	29.6 t	29.7 t	28.1 t		*α* 1.70, *β* 0.99 ^a^	29.9 t
22	*α* 1.60, *β* 1.76 ^a^	34.1 t	34.3	32.5 t		*α* 1.61 dt (13.0, 3.4)	33.9 t
						*β* 1.78 td (13.0, 3.4)	
23	1.01 s	28.0 q	28.1 q	27.4 q		*α* 4.04 d (9.8)	75.2 t
						*β* 4.52 d (9.8)	
24	1.18 s	16.0 q	16.2 q	15.3 q		1.01 s	14.3 q
26	0.80 s	18.2 q	18.3 q	17.4 q		0.92 s	21.1 q
27	1.43 s	25.8 q	25.9 q	25.8 q		1.45 s	26.5 q
28		ND	184.6 s ^a^	ND ^b^			184.0 s
29	0.93 s	28.8 q	29.0 q	27.7 q		0.94 s	28.7 q
30	0.97 s	25.2 q	25.3 q	24.5 q		0.98 s	25.4 q

^a^ Assignments based on 2D experiments. ^b^ Not detected. ^c^ overlapped.

**Figure 1:**
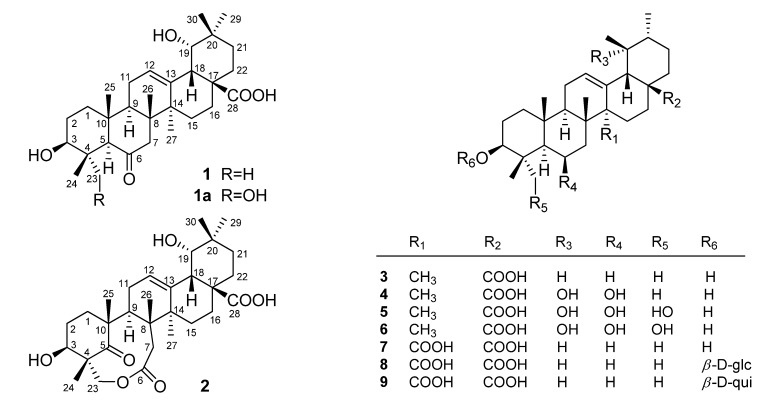
The chemical structures of compounds **1**−**9**.

The remaining 2D NMR (COSY, HSQC, HMBC and ROESY) data were consistent with the structure depicted in Figure 1 for **1**. These 1D and 2D observations, when combined with the molecular formula C_30_H_46_O_5_, suggested the presence of a 28-carboxylic group. Compound **1** was therefore determined to be 3*β*, 19*α*-dihydroxy-6-oxo-olean-12-en-28-oic acid, and named uncarilic acid.

**Figure 2:**
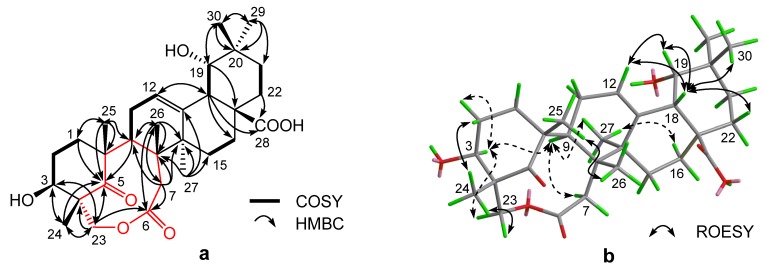
^1^H-^1^H-COSY and selected HMBC correlations of 2 (**a**); Selected key ROESY correlations of 2 (**b**).

Compound **2** was obtained as a white powder. Its molecular formula was deduced to be C_30_H_44_O_7_ from the HREI-MS peak at *m/z* 516.3096 ([M]^+^; calcd. for 516.3087) and confirmed by ^13^C-NMR and DEPT data (Table 1). The IR spectrum of **2** exhibited characteristic bands at 3,441 (OH), 1,701 (C=O) and 1,628 (olefinic C=C) cm^−^^1^. The ^1^H-NMR of **2** exhibited six singlet methyls at *δ* 0.92, 0.94, 0.98, 1.01, 1.08 and 1.45, one broad singlet at *δ* 3.16, signals assignable to two methine protons bearing a hydroxyl group at *δ* 3.28 (br d, *J* = 3.7 Hz) and 4.94 (t, *J* = 9.4 Hz), two pairs of doublets with large coupling constants due to two methylenes at *δ* 4.04, 4.52 (each 1H, d, *J* = 9.8 Hz) and *δ* 2.52, 2.30 (each 1H, d, *J* = 19.1 Hz), and one signal of an olefinic proton at *δ* 5.47 (br t). In the ^13^C-NMR spectrum (CD_3_OD), the thirty carbons were resolved as six methyls (*δ* 28.7, 26.5, 25.4, 21.4, 21.1, 14.3), nine *sp*^3^ methylenes (*δ* 75.2, 42.2, 33.9, 32.9, 31.1, 29.9, 29.1, 27.3, 26.2), four *sp*^3^ methines (two oxygenated at *δ* 82.4 and 69.3), six *sp*^3^ quaternary carbons, two *sp*^2^ carbons of a trisubstituted olefin (*δ* 144.8 and 125.4), a carboxyl (*δ* 184.0), a carbonyl (*δ* 220.9), and an ester (*δ* 177.1). Comparison of the above data to those of 3*β*,19*α*,23-trihydroxy-6-oxo-olean-12-en-28-oic acid (**1a**) [8] showed that compound **2** differed from **1a** mainly in the absence of the CH-5 and presence of an ester group, which suggested that compound **2** was possibly a 3*β*,19*α*,23-trihydroxy oleanane-type triterpenoid acid with variations occurring around C-5. The HMBC correlations (Figure 2a) from H_2_-1, H-9, H_3_-24 and H_3_-25 to the carbonyl carbon (*δ*_C_ 220.9) indicated that the ketone unit was located at C-5 rather than C-6 (as in **1a**), while HMBC correlations from H_2_-23 to C-3, C-4, C-5 and C-24 indicated that the oxymethylene (*δ*_H_ 4.04, 4.52; *δ*_C_ 75.2) were C-23. The HMBC correlations from H_2_-23 and H_2_-7 to the ester carbonyl (*δ*_C_ 177.1) indicated that CH_2_-7 was connected to CH_2_-23 *via* the C-6 ester group. Thus a nine-membered keto-lactone ring, oxonane-2,7-dione, drawn with a red bond was established. Further analysis of the remaining 2D-NMR data suggested that compound **2** had the planar structure as depicted in Figure 2a. The stereochemistry of **2** was determined by ROESY correlation analysis (Figure 2b). H-3*α* is supported by its correlations with H-9 and H-2*α*. The oxymethylene (CH_2_-23) at C-4 was *α*-oriented as it correlated with H-3. The remaining 2D NMR data were consistent with the structure depicted in Figure 1 for compound **2**, which was named secouncarilic acid.

**Scheme 1:**
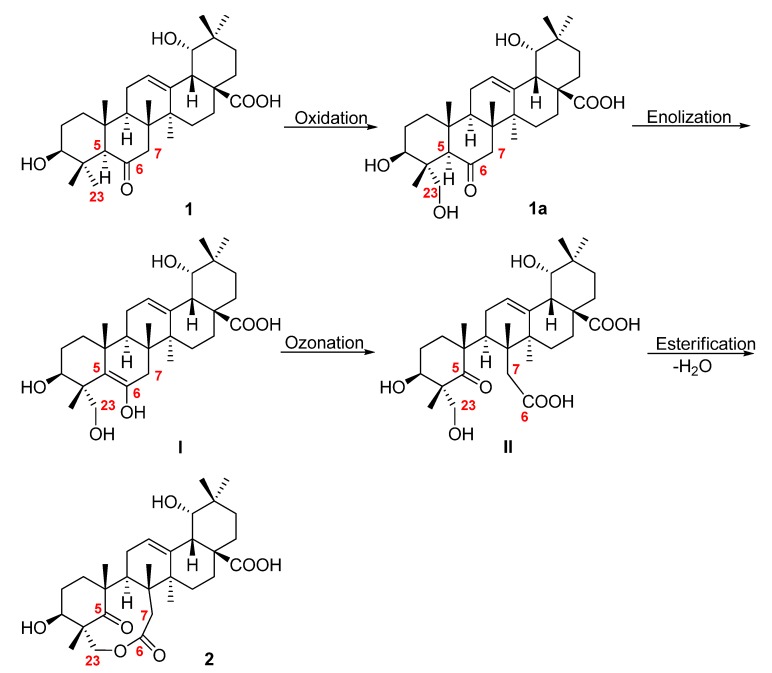
Plausible biogenetic route of **1** to **2**.

The structures of the seven ursane-type triterpenoids, ursolic acid (**3**) [9], 3*β*,6*β*,19*α*-trihydroxyurs-12-en-28-oic acid (**4**) [10], 3*β*,6*β*,19*α*-trihydroxy-23-oxo-urs-12-en-28-oic acid (**5**) [10], 3*β*,6*β*,19*α*,23- tetrahydroxyurs-12-en-28-oic acid (**6**) [11], quinovic acid (**7**) [9], cinchonaglycoside C (**8**) [12] and 3-O-[*β*-D-quinovopyranosyl] quinovic acid (**9**) [13] were determined by comparing their spectroscopic data to those reported in the literature. To the best of our knowledge, this is the first report of non-alkaloids isolated from *Uucaria sessilifructus* Roxb. These oleanane- and ursane-type triterpenoids are also of chemotaxonomic significance as ursane- or oleanane-type triterpenoids were reported to be the characteristic metabolites of the genus *Uncaria* [4,5]. In addition, uncarilic acid is the second 6-oxo oleanane-type triterpenoid ever reported, while secouncarilic acid is the first oleanane-type 5,6-secotriterpenoid. As show in Scheme 1, the biogenetic origin of secouncarilic acid (**2**) could be traced back to uncarilic acid (**1**). Oxidation of the CH_3_-23 of **1** to a hydroxymethyl produced **1a**, enolization of the carbonyl-6 of which followed by ozonation of the newly generated intermediate I would yield II. In the final step, esterification between the 23-hydroxy and the 6-carboxylic group of II would occur to give **2**. 

Compounds **1**–**9** were evaluated for their inhibitory activities against nitric oxide production in LPS-activated RAW264.7 macrophages according to the method [14]. Results showed all the compounds tested did not show any obvious inhibitory activity (IC_50_ > 25 μM).

## 3. Experimental

### 3.1. General

Optical rotations were measured on a Perkin-Elmer 24173 polarimeter. IR spectra were measured using a Bruker Tensor 27 instrument with KBr disc; HREI-MS were carried out on an AutoSpec Premier P776 spectrometer. 1D and 2D-NMR spectra were recorded on a Bruker DRX-500 and Bruker Avance III 600 spectrometer with TMS as internal standard. Silica gel (200–300 mesh, Qingdao Marine Chemical, Qingdao, P.R. China) was used for column chromatography. Semi-preparative HPLC was performed on an XTerra prep RP-18 (10 μm, Waters Corp., Wexford, Ireland) column (10 × 250 mm) eluted with MeOH/H_2_O from 50:50 to 90:10 for 15 min at a flow rate of 4 mL/min; the detector used was PDA (200–400 nm) at 33 °C. Fractions were monitored by TLC, and spots were visualized by spraying TLC plates with 10% sulfuric acid in ethanol and heating at 110 °C for 5–10 min.

### 3.2. Plant Material

The hooks and stems of *Uucaria sessilifructus* were collected in Xishuangbanna, Yunnan Province, China, in August, 2011 and authenticated by one of the authors (Y-K Xu) of the Xishuangbanna Tropical Botanical Garden. Voucher specimens of the *U. sessilifructus* (No. 143757) have been deposited in the Herbarium of the Xishuangbanna Tropical Botanical Garden, Chinese Academy of Sciences.

### 3.3. Extraction and Isolation

The air-dried and powdered hooks and stems of *U. sessilifructus* (9.0 kg) were extracted three times (each for 6 days) with 95% ethanol in water at room temperature. The extract was filtered and concentrated under reduced pressure until only H_2_O remained. The remaining solution was adjusted to pH 3 using 10% H_2_SO_4_ and then extract with EtOAc to give EtOAc and water extracts after concentration. The EtOAc extract (310 g) was then subjected to silica gel column chromatography (CC) eluted with petroleum ether/EtOAc (from 50:1 to 0:1) through EtOAc/MeOH (from 10:1 to 3:1) to yield five major fractions (1–5). Fraction 3 (5.8 g) was subjected to silica gel CC (CHCl_3_/MeOH, 50:1→10:1) to give **3** (29 mg) and **4** (8 mg). Fraction 4 (28.4 g) was subjected to silica gel CC (CHCl_3_/MeOH, 20:1→3:1) to give **5** (11 mg), **6** (23 mg) and three major fractions (Fr. 1-1–Fr. 1-3). Fraction 1-2 (3.0 g) was purified by semi-preparative HPLC (MeOH/H_2_O, 50/50→90/10; flow rate: 4 mL/min) to give **1** (12 mg), **2** (7 mg) and **7** (17 mg), fraction 1–3 (2.2 g) was purified by semi-preparative HPLC (MeOH/H_2_O, 60/40→90/10; flow rate: 4 mL/min) to give **8** (16 mg) and **9** (13 mg).

### 3.4. Spectral Data

Uuncarilic acid (**1**). White amorphous powder; 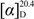 +19.7 (MeOH; *c* 0.18); IR (KBr) ν_max_ (cm^−1^): 3441, 2932, 2871, 1702, 1630, 1562, 1554, 1458, 1392, 1283, 1253, 1207, 1171, 1156, 1134, 1109, 1090, 1051, 1031, 986; HREI-MS (*m/z* 486.3345, [M]^+^; calcd. for 486.3356); ^1^H- and ^13^C-NMR data: see Table 1. 

Secouncarilic acid (**2**). White amorphous powder; 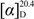 +34.8 (MeOH; *c* 0.19); IR (KBr) ν_max_ (cm^−1^): 3441, 2937, 2876, 1720, 1701, 1655, 1628, 1457, 1385, 1347, 1302, 1258, 1233, 1209, 1165, 1134, 1070, 1048, 1014, 982; HREI-MS (*m/z* 516.3096, [M]^+^; calcd. for 516.3087); ^1^H- and ^13^C-NMR data: see Table 1.

### 3.5. Inhibition of NO Production in LPS-Stimulated RAW 264.7 Macrophage Cell Line

The assay was performed according to a previously described method [13]. Each compound was dissolved in DMSO and further diluted in the medium to produce different concentrations with a maximum concentration of 25 μM. The absorbance was measured at 570 nm with a 2104 Envision Multilabel Plate Reader (Perkin-Elmer Life Sciences, Inc., Boston, MA, USA). Cytotoxicity was determined with the MTT assay. MG-132 (Sigma-Aldrich, Foster City, CA USA) was used as the positive control.

## 4. Conclusions

In summary, one new 6-oxo oleanane-type triterpenoid, uncarilic acid, and its 5,6-secotriterpenoid derivative, secouncarilic acid, together with seven ursane-type triterpenoids, including four ursolic acid derivatives and three quinovic acid derivatives, were isolated from the hooks and stems of *Uucaria sessilifructus*. Secouncarilic acid is the first oleanane-type 5,6-secotriterpenoid, while uncarilic acid is the second 6-oxo oleanane-type triterpenoid ever reported. A plausible biogenetic route from uncarilic acid to secouncarilic acid was also postulated. None of the nine compounds exhibited significant inhibitory activities against nitric oxide production in LPS-activated RAW264.7 macrophages.

## Supplementary Materials

Supplementary materials can be accessed at: http://www.mdpi.com/1420-3049/18/8/9727/s1.
