# Supplementary material for: Uncarilic Acid and Secouncarilic Acid, Two New Triterpenoids from Uucaria sessilifructus
Source: Molecules. 2013 Aug 14;18(8):9727–34. doi: 10.3390/molecules18089727 (PMC6270339; doi:10.3390/molecules18089727)
Supplement: Supplementary file 1 [file molecules-18-09727-s001.pdf]

Supporting Information

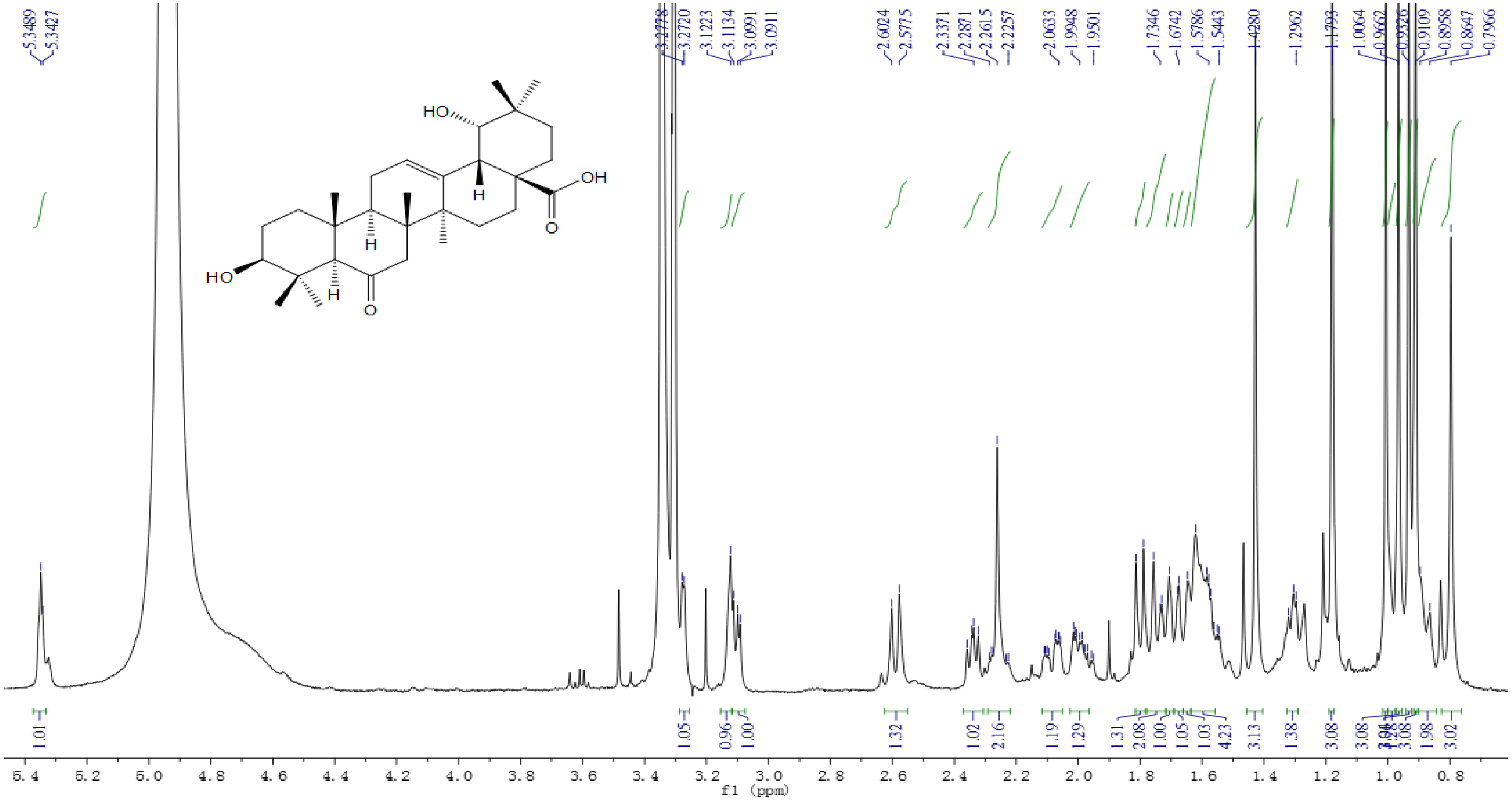

Figure S1. ^{1}H-NMR spectrum of uncarilic acid (**1**) at 500MHz in CD_3OD.

Figure S2. ^{13}C-NMR spectrum of uncarilic acid (1) at 125 MHz in CD_3OD.

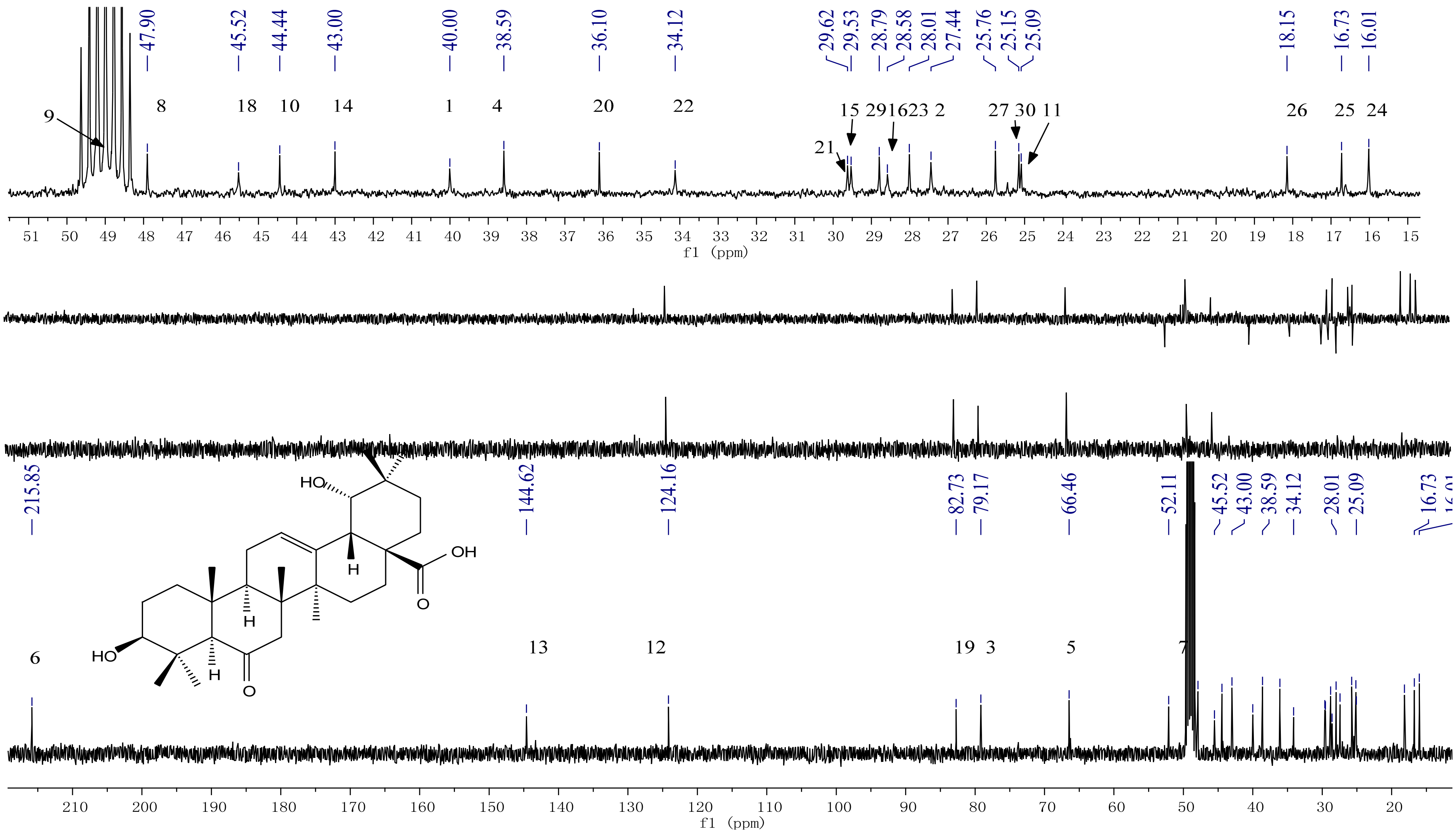

Figure S2-1. ^{13}C-NMR spectrum of uncarilic acid (**1**) at 150 MHz in CD_3OD.

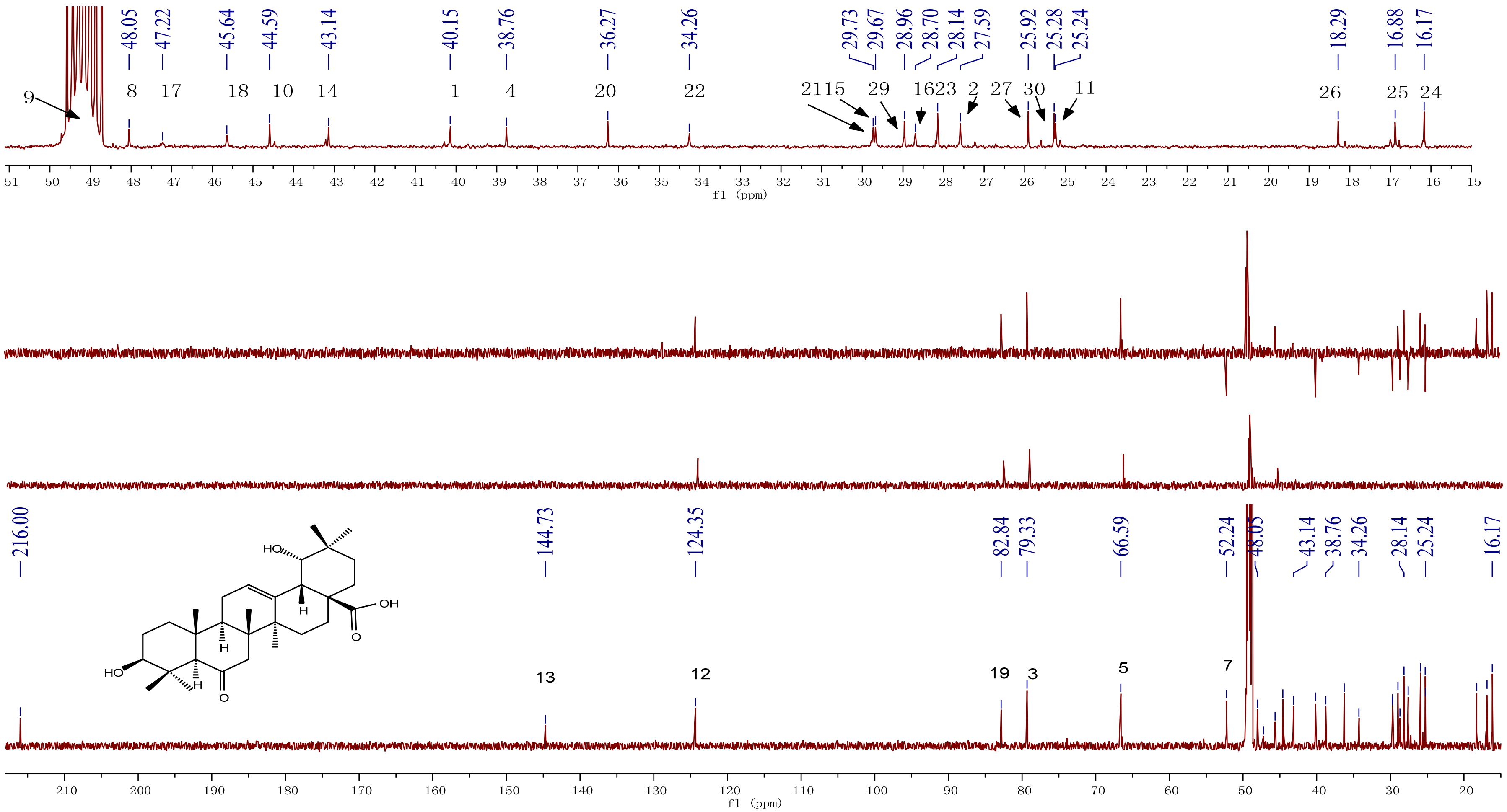

Figure S2-2. ^{13}C-NMR spectrum of uncarilic acid (**1**) at 150 MHz in $CDCl_3$.

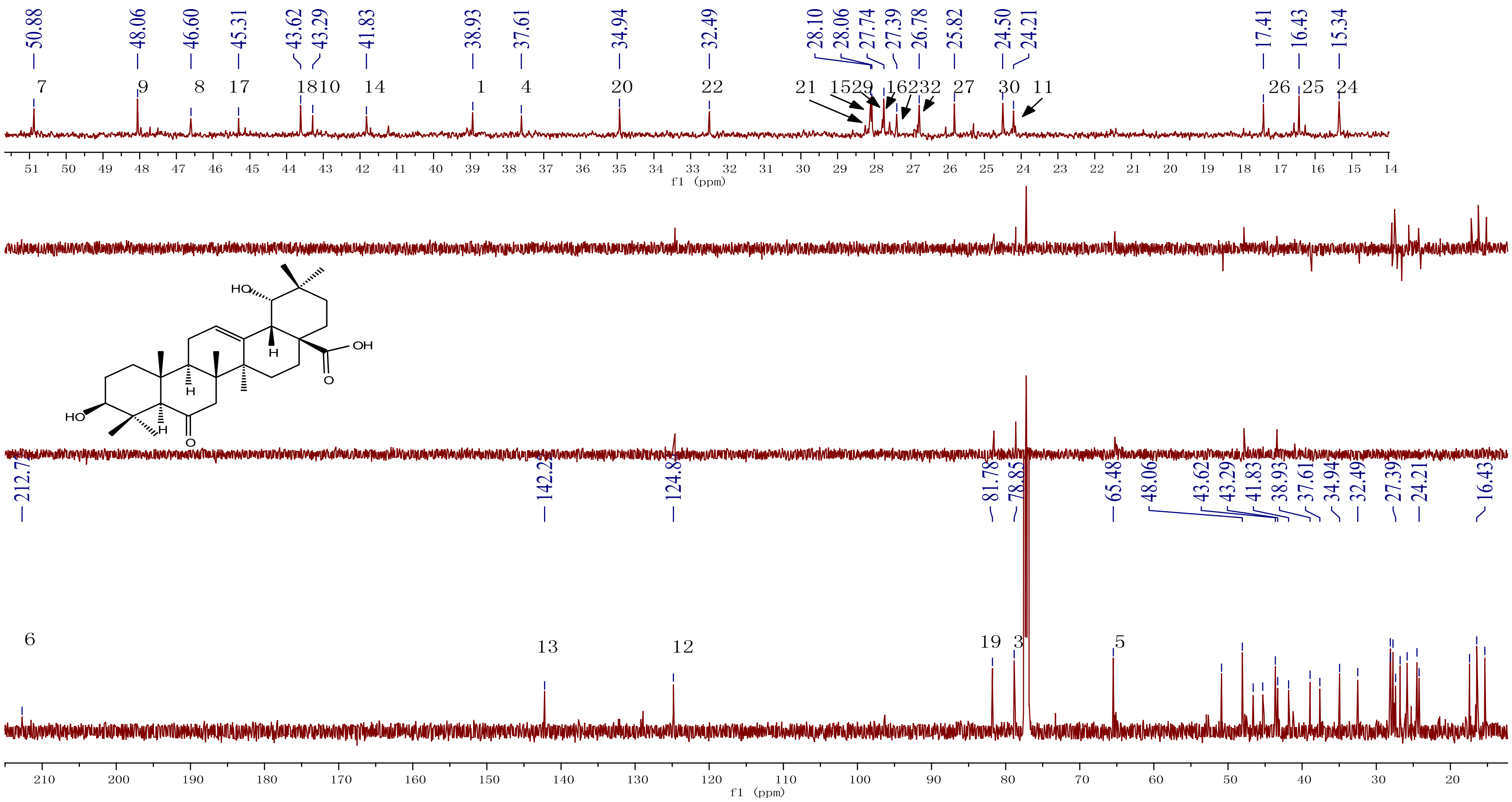

Figure S3. HSQC spectrum of uncarilic acid (1) at 500/125 MHz in CD_3OD.

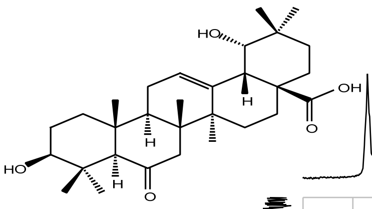

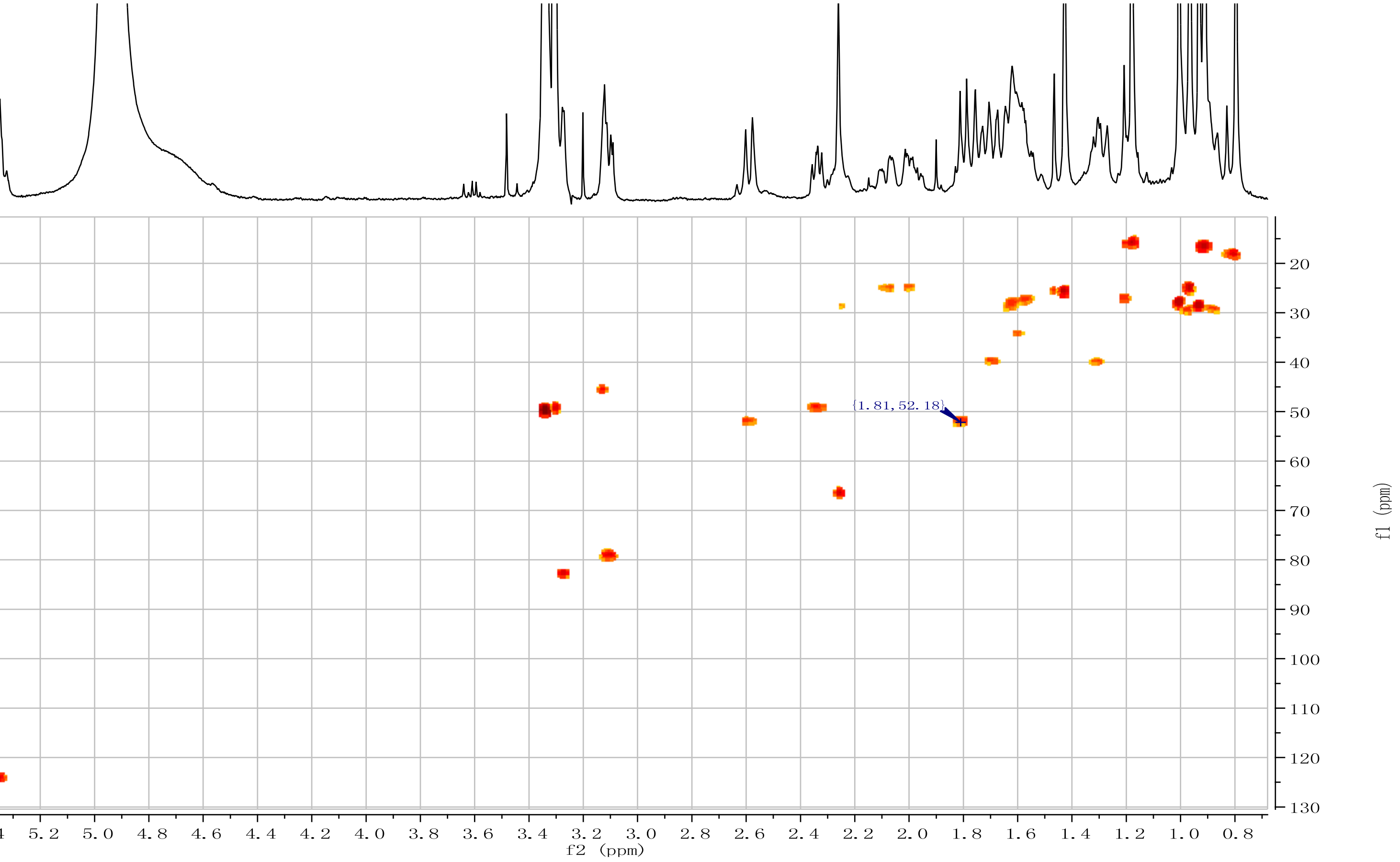

Figure S4. COSY spectrum of uncarilic acid (**1**) at 500 MHz in CD_3OD.

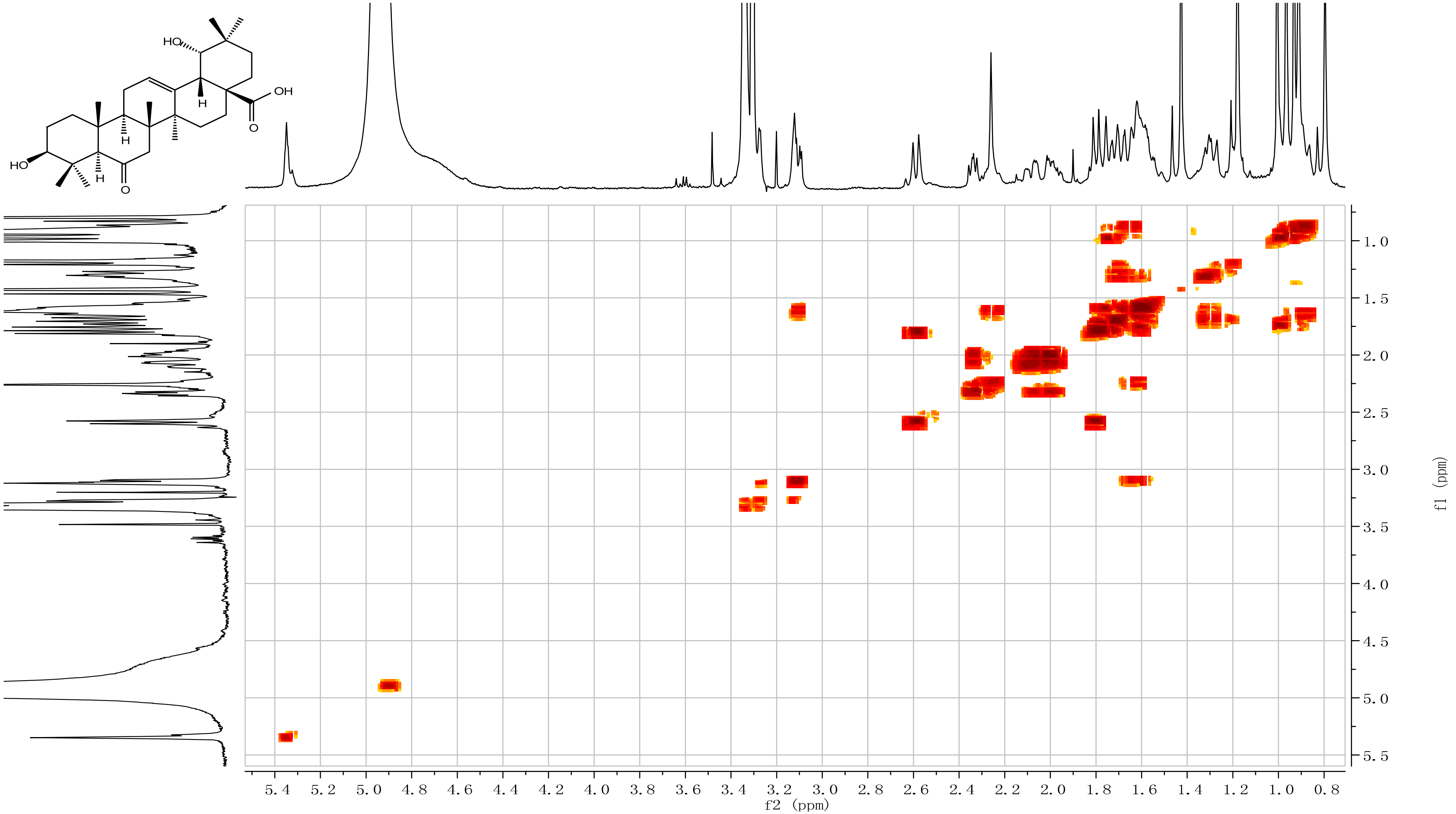

Figure S5. HMBC spectrum of uncarilic acid (**1**) at 500/125MHz in CD_3OD.

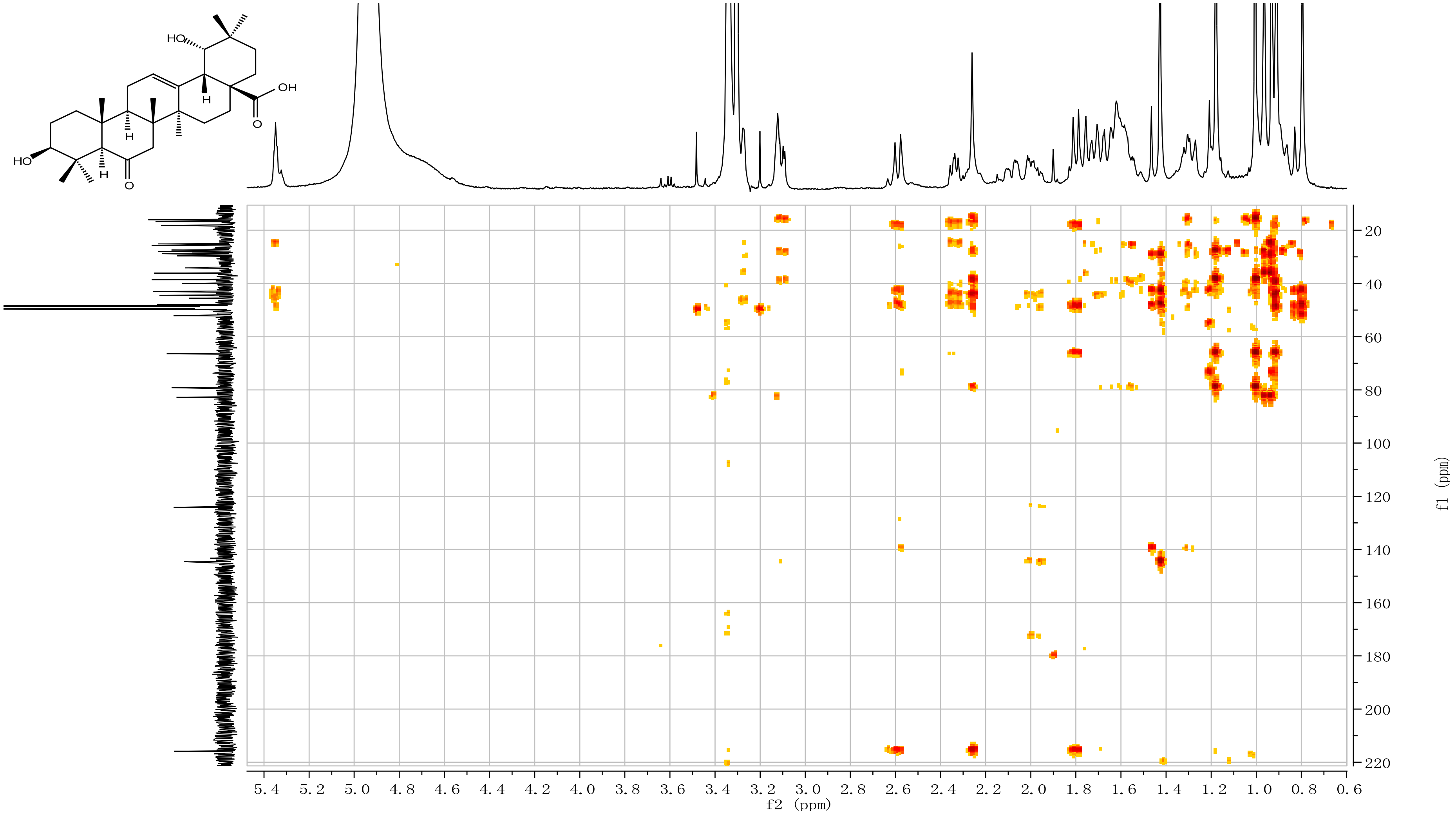

Figure S5-1. HMBC spectrum of uncarilic acid (**1**) at 600/150 MHz in CD_3OD.

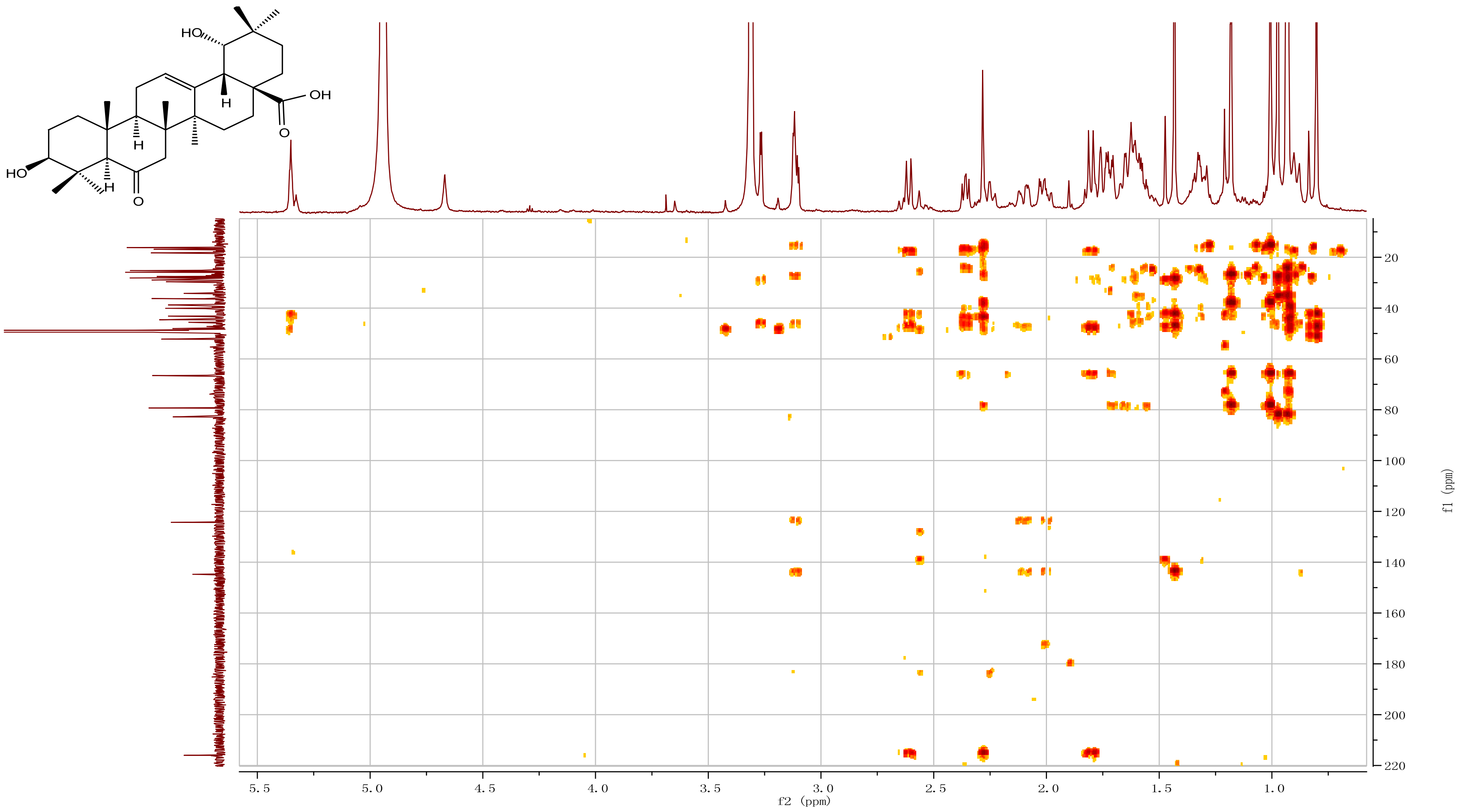

Figure S5-2. Key correlations H-9/C-25 and H-7/C-9,H_3-26/C-9 of HMBC (**1**) at 600/150MHz in CD_3OD.

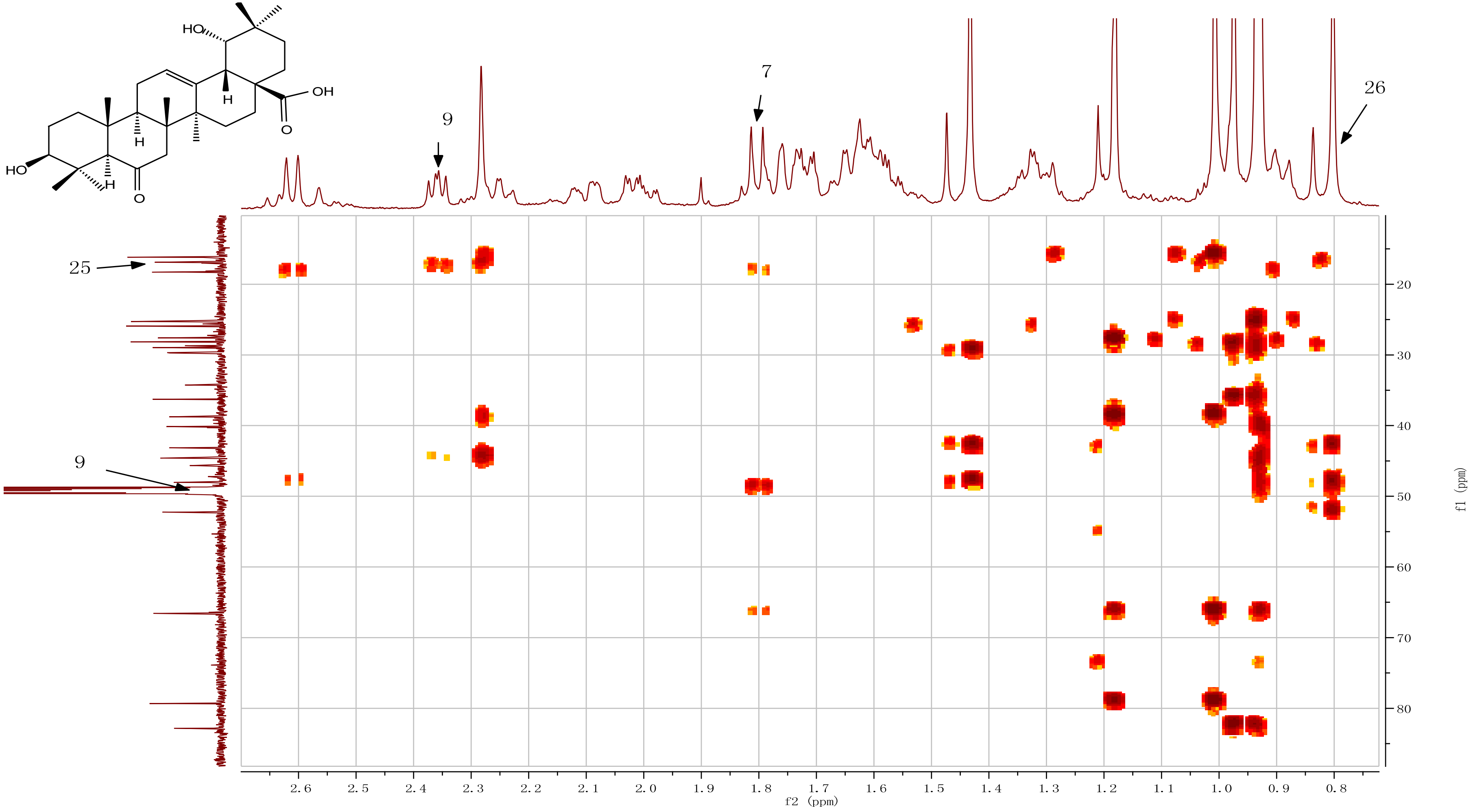

Figure S5-3. Key correlations H-16/C-28 of HMBC(**1**) at 600/150 MHZ in CD_3OD.

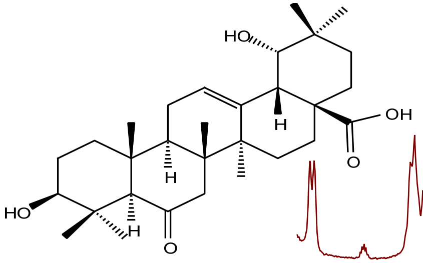

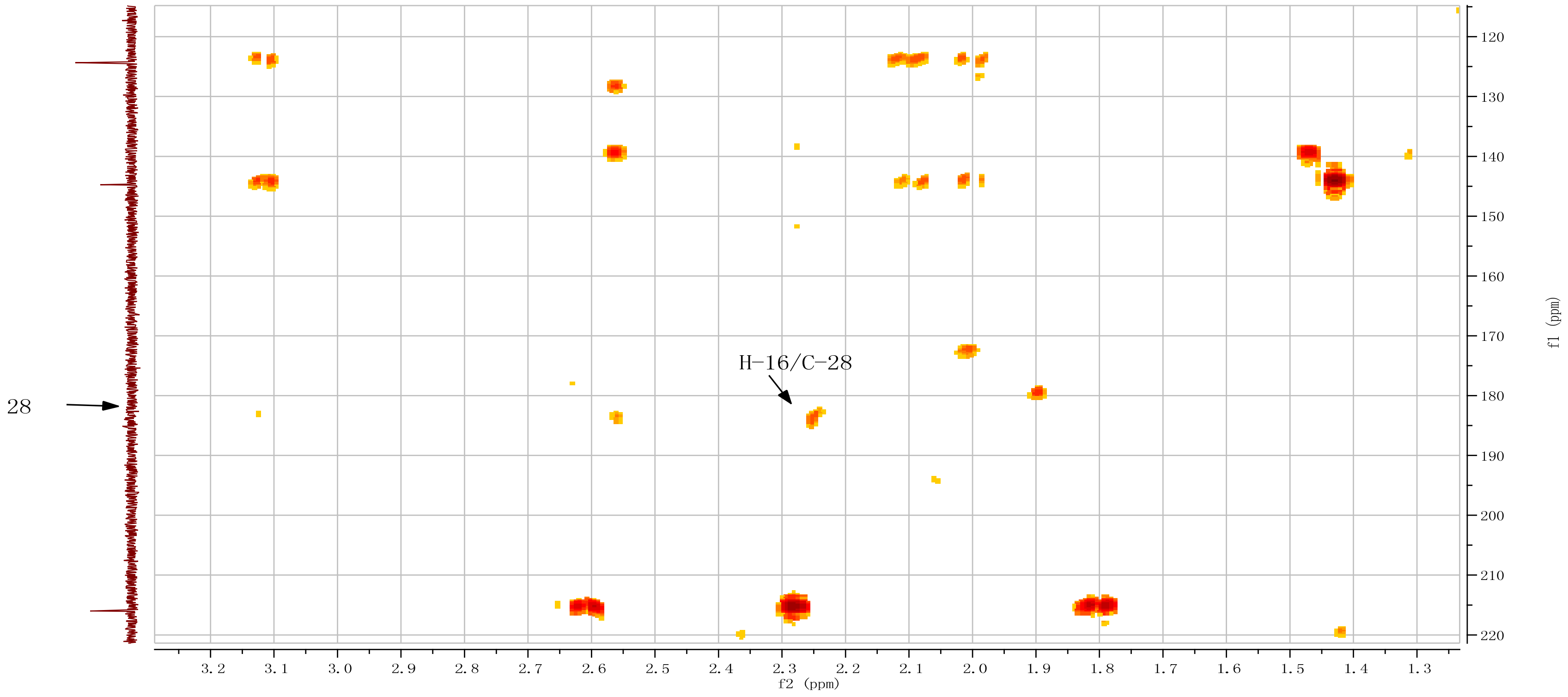

Figure S6. ROESY spectrum of uncarilic acid (**1**) at 500 MHz in CD_3OD.

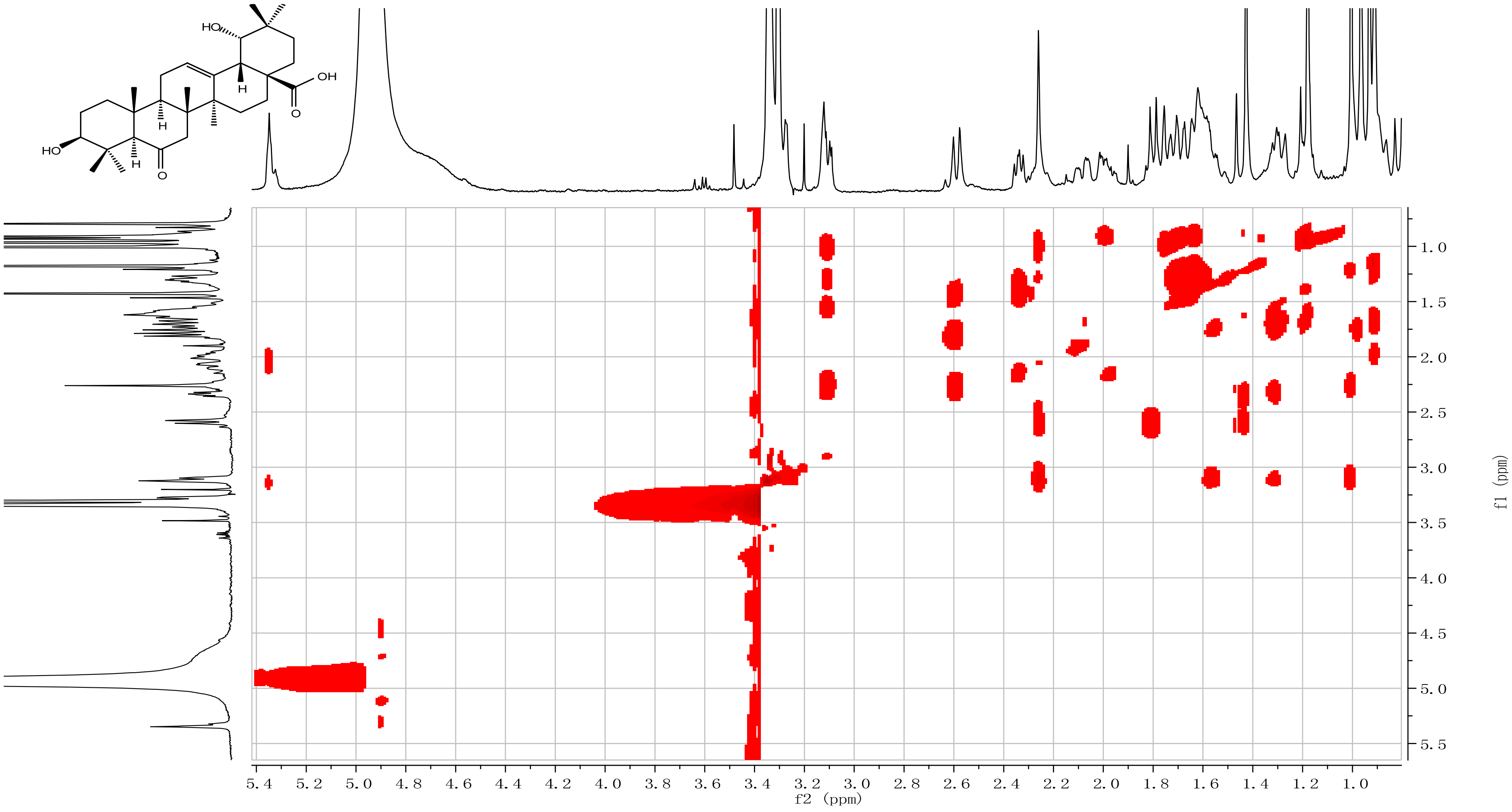

Figure S7. HREI-MS spectrum of uncarilic acid (**1**).

Elemental Composition Report **Page 1**

Single Mass Analysis
Tolerance = 10.0 PPM / DBE: min = -10.0, max = 120.0
Selected filters: None

Monoisotopic Mass, Odd and Even Electron Ions
21 formula(e) evaluated with 1 results within limits (up to 51 closest results for each mass)
Elements Used:
C: 0-200 H: 0-400 O: 4-6

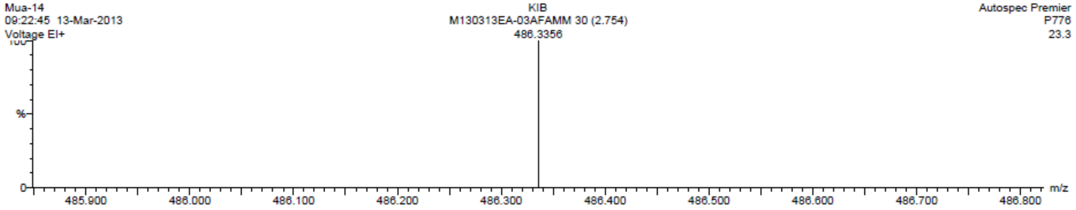

Minimum:				-10.0		
Maximum:		200.0	10.0	120.0		
Mass	Calc. Mass	mDa	PPM	DBE	i-FIT	Formula
486.3356	486.3345	1.1	2.3	8.0	5546031.5	C30 H46 O5

Figure S8. IR spectrum of uncarilic acid; (**1**).

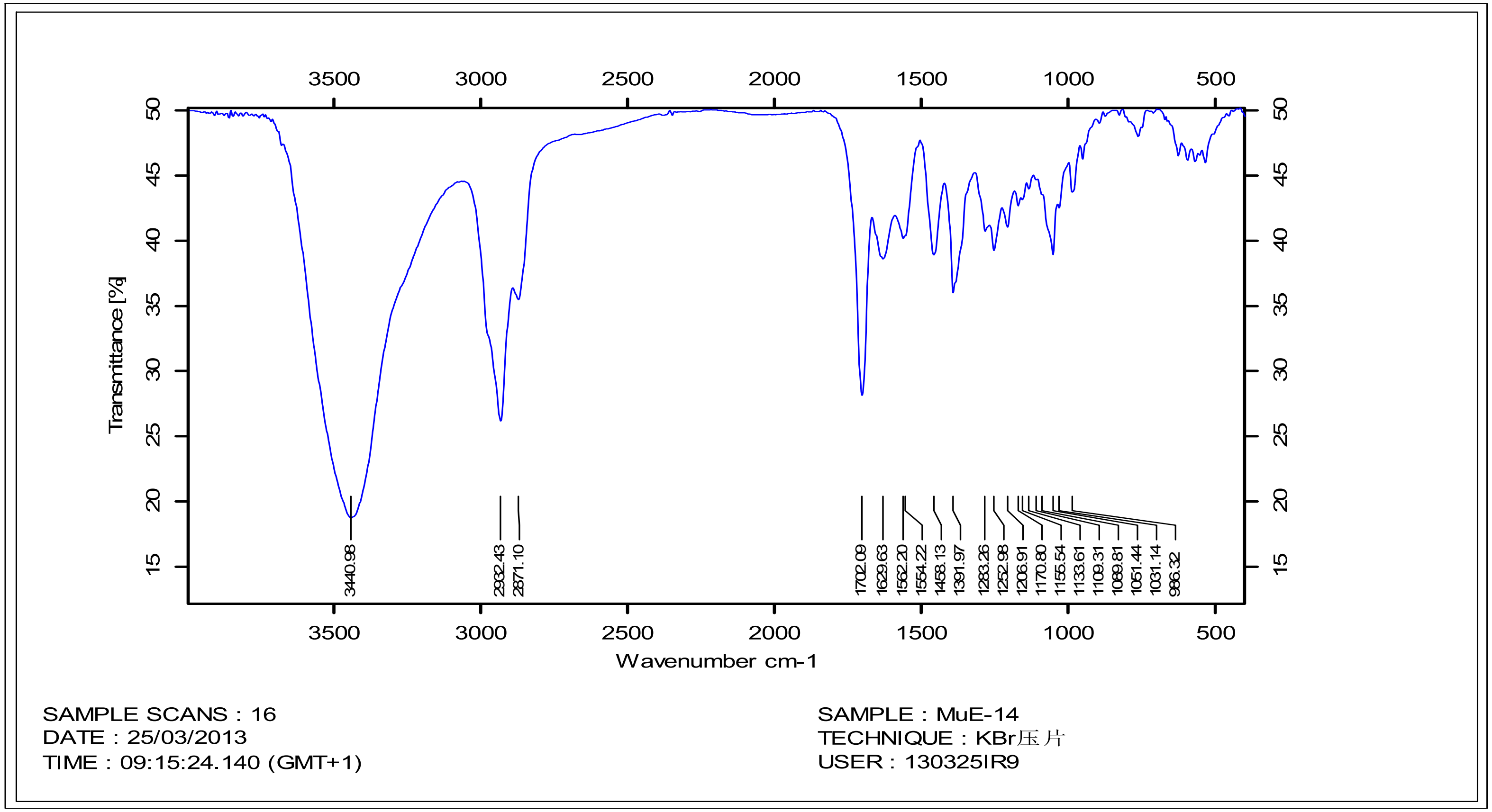

Figure S9. Optical rotation measurement of uncarilic acid (**1**)

5 (1/3)	Specific O.R.	19.000	20.4	0	Mon Mar 25 13:06:46 2013	0.00180g/mlMeOH	MUE-14
5 (2/3)	Specific O.R.	20.556	20.4	0	Mon Mar 25 13:07:00 2013	0.00180g/mlMeOH	MUE-14
5 (3/3)	Specific O.R.	19.444	20.4	0	Mon Mar 25 13:07:13 2013	0.00180g/mlMeOH	MUE-14

Figure S10. ^{1}H-NMR spectrum of secouncarilic acid (**2**) at 500 MHz in CD_3OD.

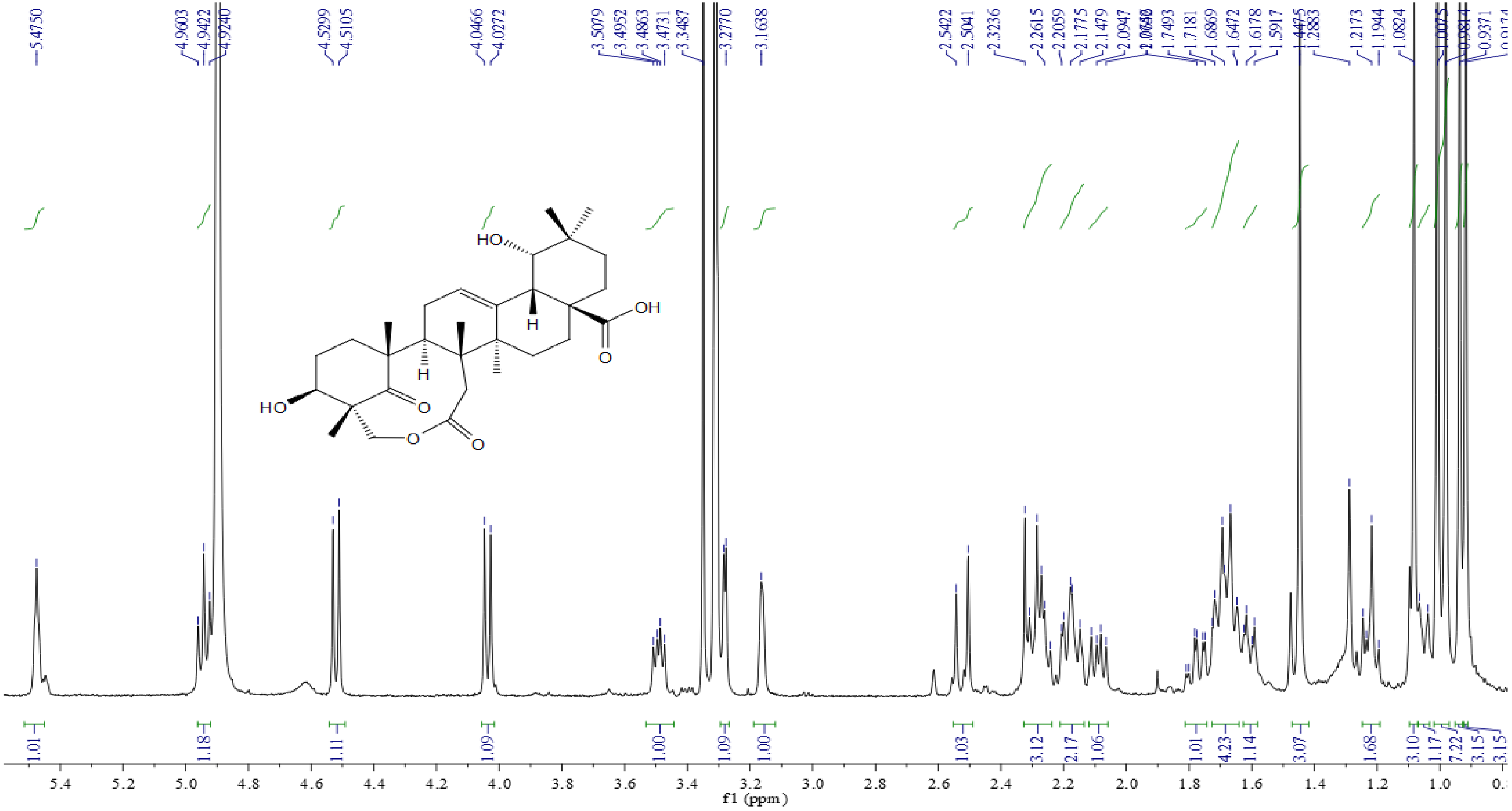

Figure S11. ^{13}C-NMR of spectrum of secouncarilic acid (**2**) at 125 MHz in CD_3OD.

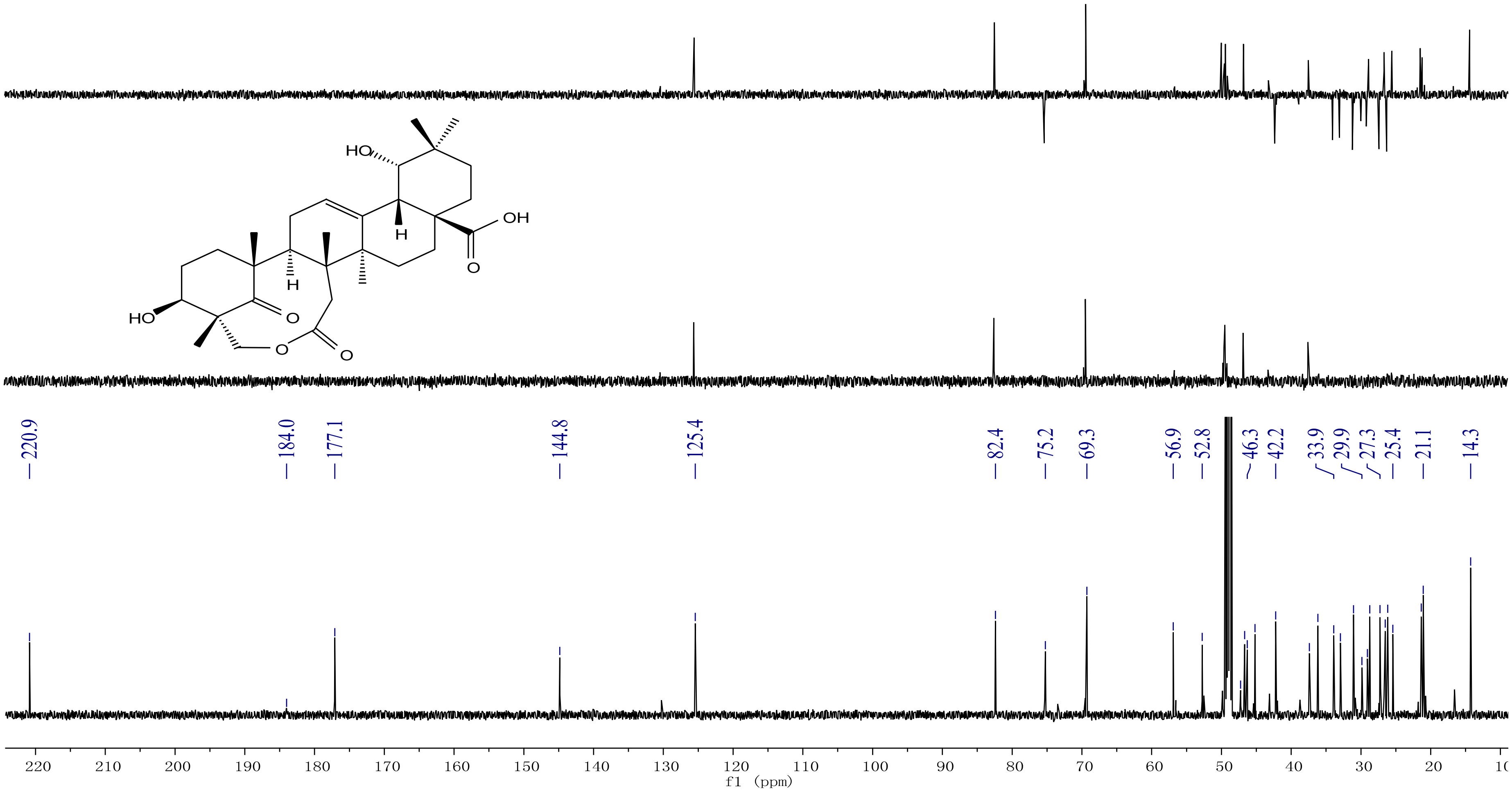

Figure S12. HSQC spectrum of secouncarilic acid (**2**) at 500/125 MHz in CD_3OD.

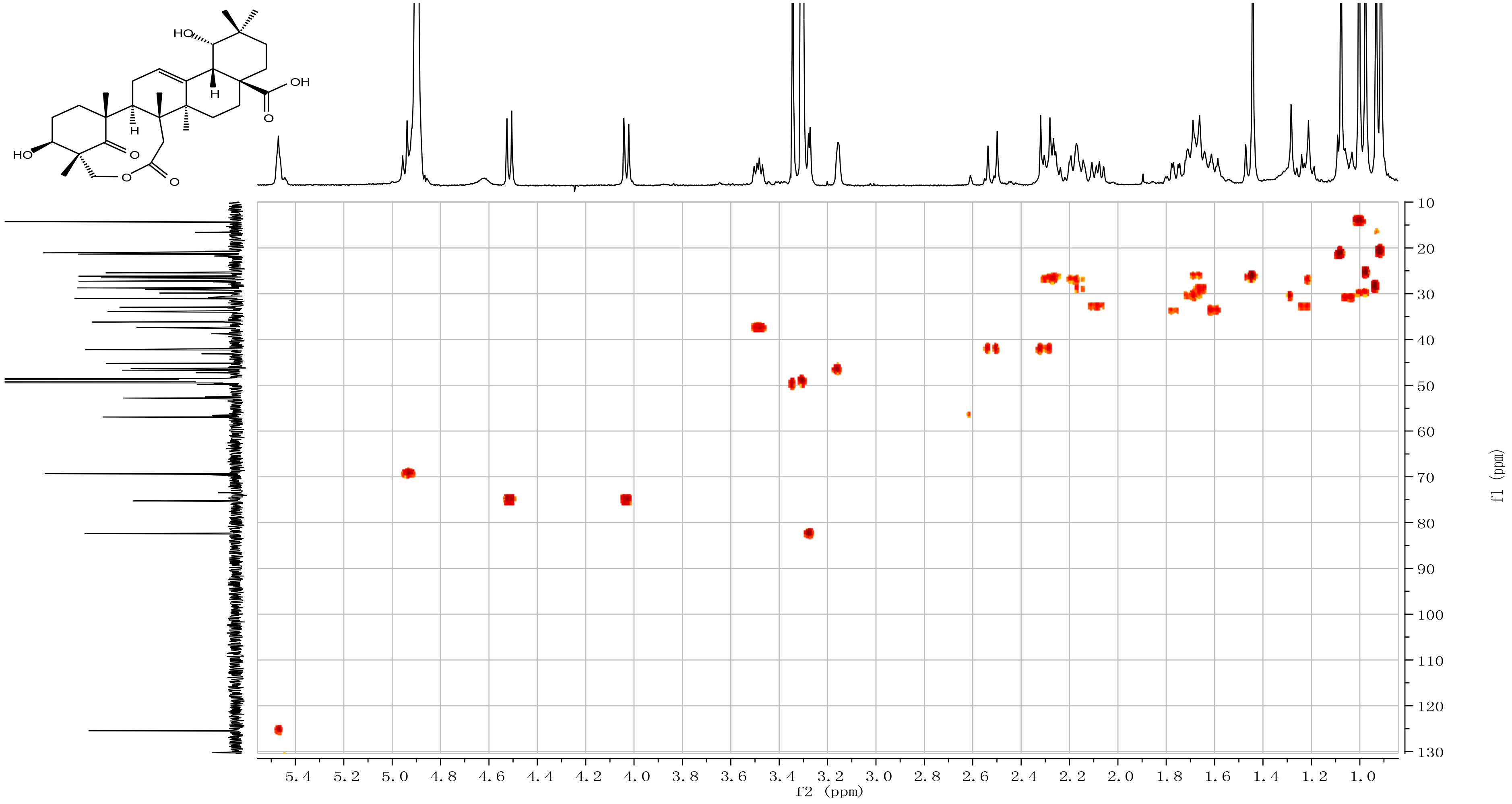

Figure S13. COSY spectrum of secouncarilic acid (**2**) at 500 MHz in CD_3OD.

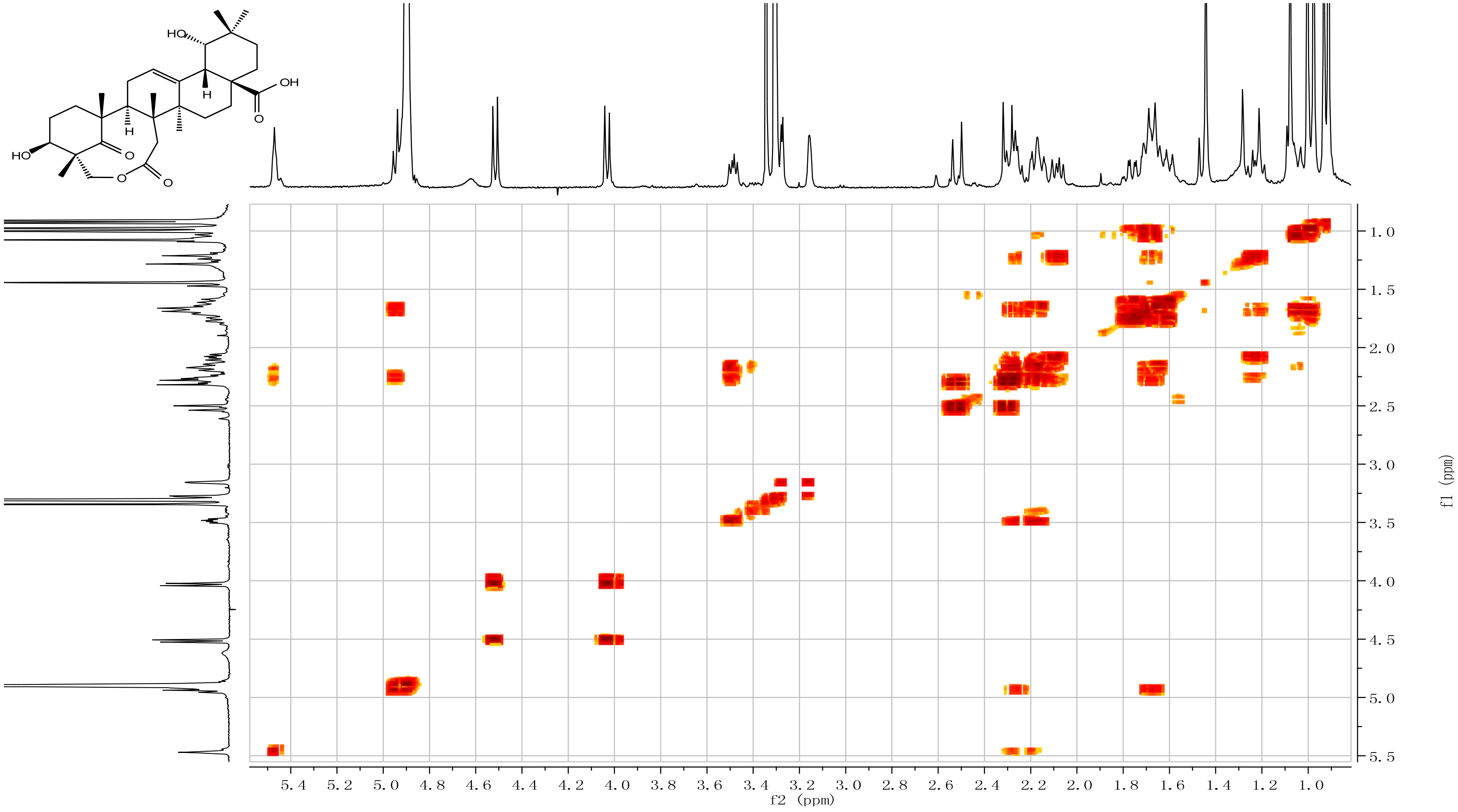

Figure S14. HMBC spectrum of secouncarilic acid (2) at 500/125 MHz in CD_3OD.

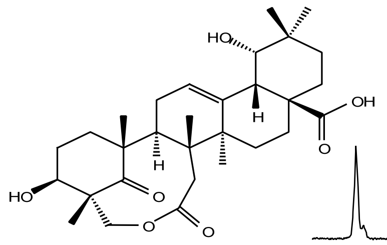

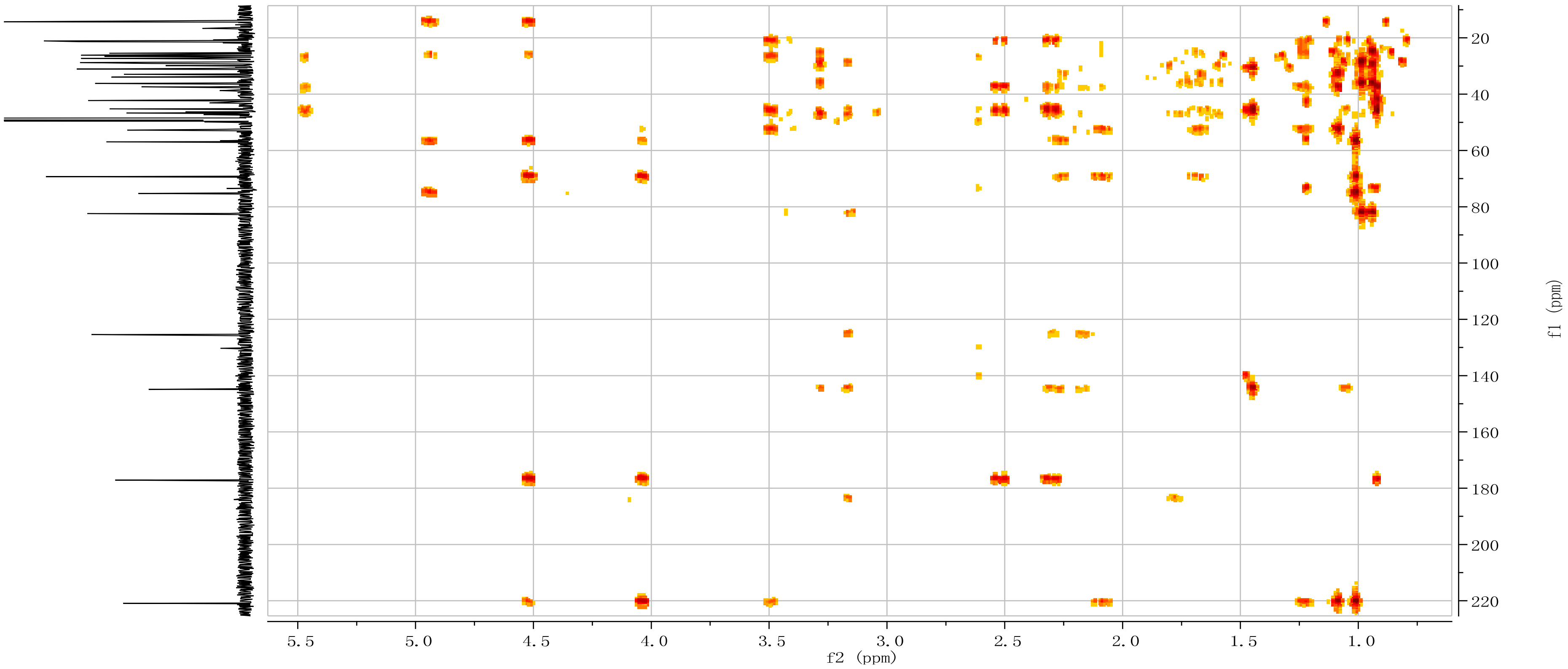

Figure S15. HSQC-TOCSY spectrum of secouncarilic acid (**2**) at 500/125 MHz in CD_3OD.

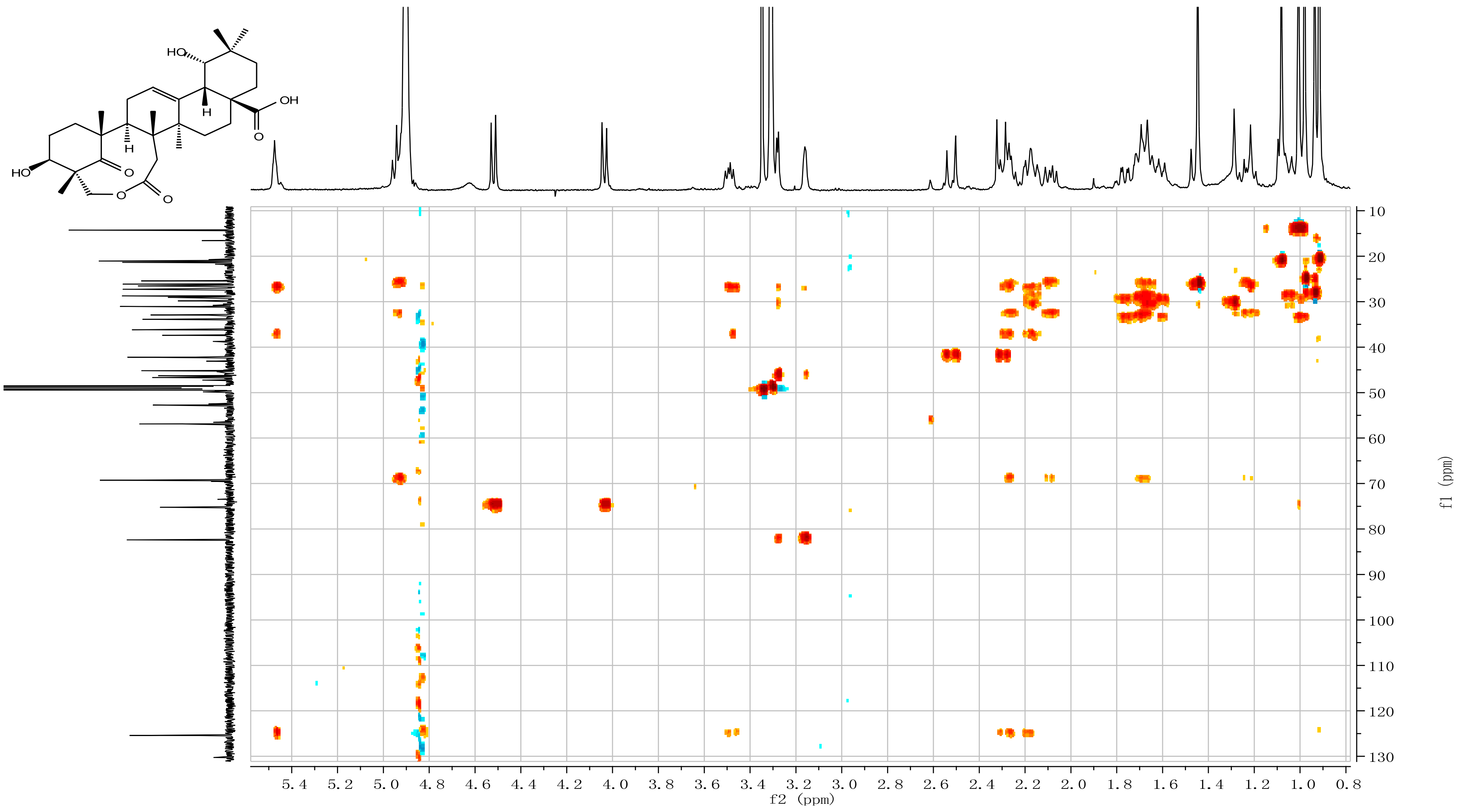

Figure S16. ROESY spectrum of secouncarilic acid (**2**) at 500 MHz in CD_3OD.

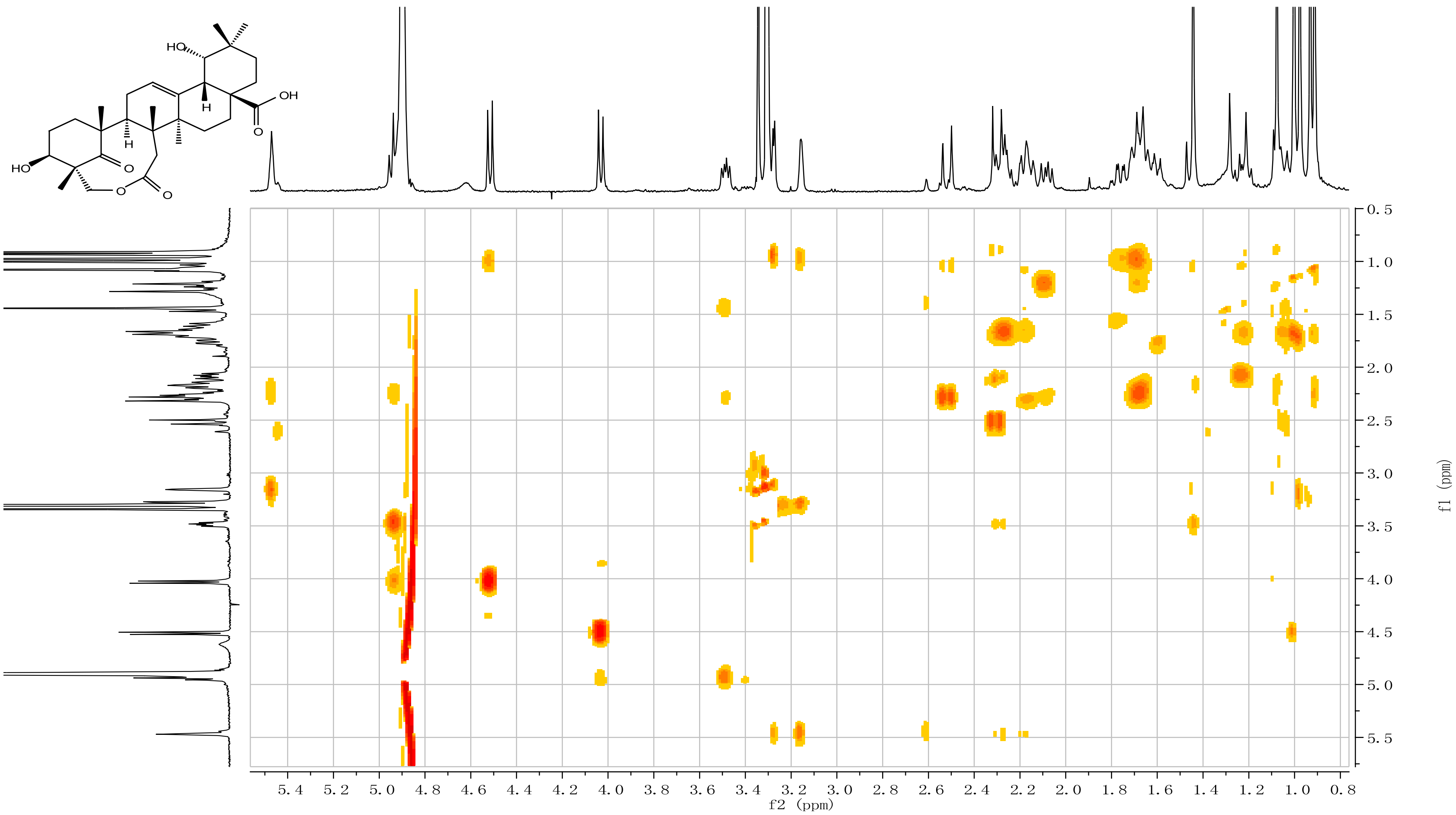

Figure S17. HREI-MS spectrum of secouncarilic acid (**2**).

Elemental Composition Report **Page 1**

Single Mass Analysis
Tolerance = 10.0 PPM / DBE: min = -10.0, max = 120.0
Selected filters: None

Monoisotopic Mass, Odd and Even Electron Ions
22 formula(e) evaluated with 1 results within limits (up to 51 closest results for each mass)
Elements Used:
C: 0-200 H: 0-400 O: 6-8

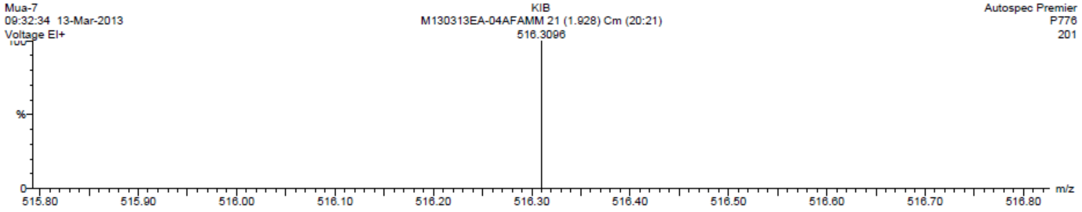

Minimum:				-10.0		
Maximum:		200.0	10.0	120.0		
Mass	Calc. Mass	mDa	PPM	DBE	i-FIT	Formula
516.3096	516.3087	0.9	1.7	9.0	5546118.5	C30 H44 O7

Figure S18. IR spectrum of secouncarilic acid (**2**).

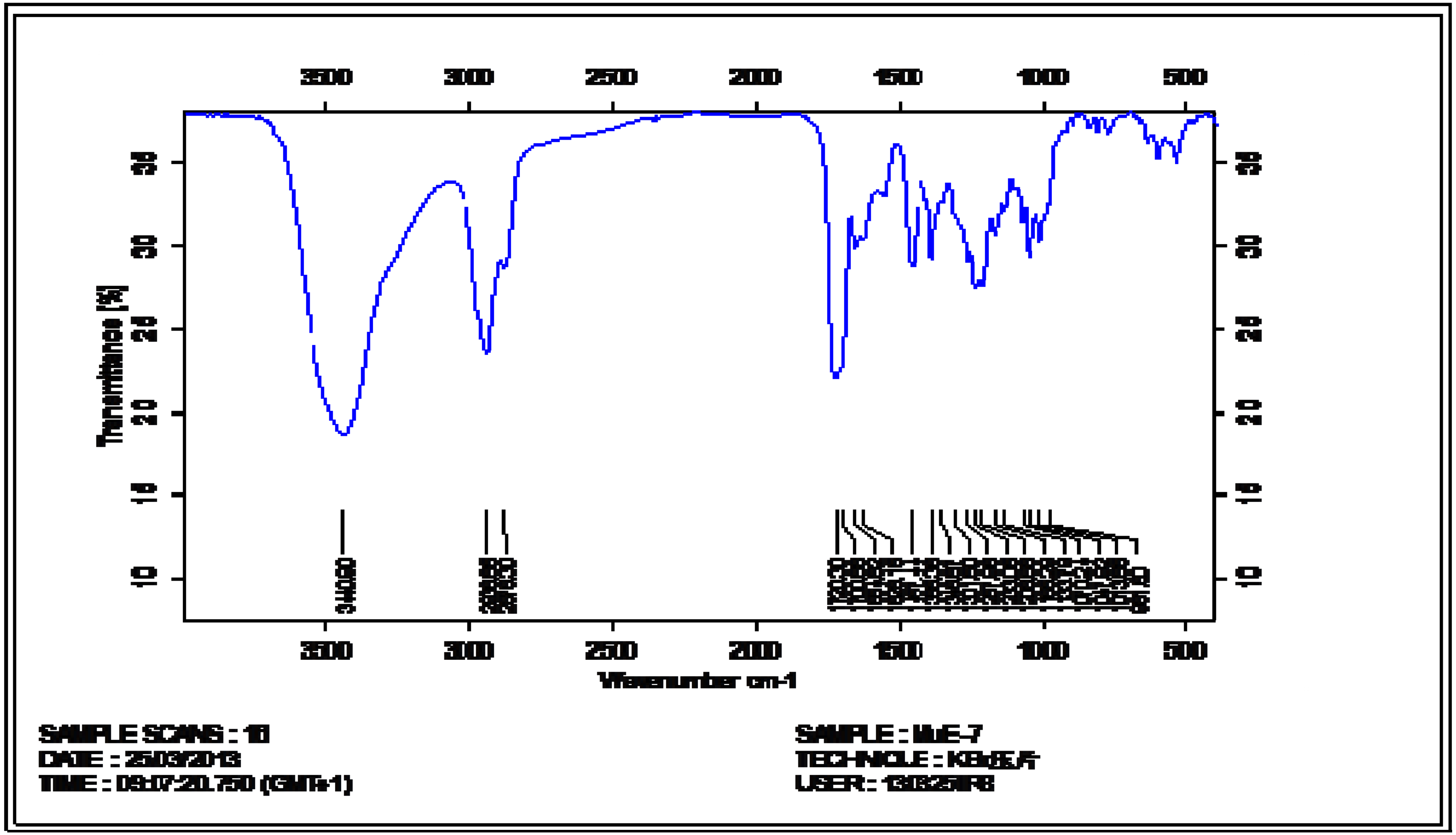

Figure S19. Optical rotation measurement of secouncarilic acid (**2**).

3 (1/3)	Specific O.R.	34.211	20.4	0	Mon Mar 25 12:56:20 2013	0.00190g/mlMeOH	MUE-7
3 (2/3)	Specific O.R.	35.053	20.4	0	Mon Mar 25 12:56:33 2013	0.00190g/mlMeOH	MUE-7
3 (3/3)	Specific O.R.	35.263	20.4	0	Mon Mar 25 12:56:46 2013	0.00190g/mlMeOH	MUE-7
